# Supplementary material for: Nonergodicity and Simpson’s paradox in neurocognitive dynamics of cognitive control
Source: Nat Commun. 2026 Apr 27;17:3494. doi: 10.1038/s41467-026-71404-0 (PMC13121725; doi:10.1038/s41467-026-71404-0)
Supplement: Supplementary file 1 — Supplementary Information [file 41467_2026_71404_MOESM1_ESM.pdf]

Supplementary Information

I. Supplementary Results

Measuring Simpson's paradox of brain networks

To further understand how Simpson's paradox (SP) affects inferences across the brain, we measured SP based on the difference between within-subjects and between-subjects inferences, in different brain networks. We defined a measure of SP as the fraction of subjects for whom the within-subjects brain-behavior association showed an opposite sign to the between-subjects association. Values above 0.5 indicate SP.

Our analysis revealed substantial variation in the extent of SP across brain networks (**SI Figure S3**). Notably, the anterior salience network consistently demonstrated the highest level of SP for all three cognitive model parameters (SSRT, probability of proactivity, and proactive delaying). The measures of SP for associations with the probability of proactivity showed wide confidence intervals, reflecting the weak between-subjects associations between this parameter and brain activation.

To understand how networks related to each other in terms of their degree of SP, we performed hierarchical clustering on the joint SP measures of the networks with respect to all three cognitive model parameters (**SI Figure S3c-d**). This analysis produced a hierarchical organization of brain networks based on their SP profiles. The anterior salience network emerged as the most dissimilar, forming its own cluster separate from all other networks. Additionally, the default mode network and retrosplenial cortex and parahippocampal gyrus clustered together, suggesting similarities in the extent to which they displayed SP.

These findings demonstrate that brain networks exhibit substantial and varying levels of SP in brain-behavior associations during this task, with the anterior salience network consistently showing the highest levels of SP. The hierarchical organization of networks by SP suggests a new perspective on the functional architecture of the brain and how it relates to cognitive control processes.

Robustness of nonergodicity to analytical choices

To test the robustness of our nonergodicity findings, we conducted several control analyses. These analyses aimed to evaluate whether nonergodicity in brain-behavior relations was specific to the brain and behavioral measures used in the main analyses.

Our primary analysis compared within-subjects and between-subjects brain-behavior associations using a canonical approach for the SST[1-6]: correlating subject-average behavioral measures with subject-average differences in brain activation between correct stop and correct go trials. However, other between-subjects analyses can also be reasonably compared against the within-subjects analyses. We tested three alternative between-subjects approaches, each correlating the subject-average behavioral measures (Go RT, SSRT, probability of proactivity, and proactive delaying) with different subject-average brain measures from the task. First, to

align more closely with our within-subjects analyses, we (i) correlated subject-average Go RT with subject-average brain activation from all Go trials (Go activation) (**Supplementary Figure S4a**), and (ii) correlated subject-average SSRT with subject-average brain activation from all stop trials (correct and incorrect combined) and subject-average probability of proactivity and proactive delaying with subject-average brain activation from all trials (go and stop combined) (**Supplementary Figure S5a**). Second, we used two other standard subject-average brain measures[4]: difference in activation between incorrect stop and correct go trials (incorrect stop versus correct go activation) (**Supplementary Figure S4b, S5b**) and difference in activation between incorrect and correct stop trials (incorrect stop versus correct stop activation) (**Supplementary Figure S4c, S5c**). Comparing these between-subjects results to the within-subjects results (**Supplementary Figure S4d, S5d**) demonstrates that nonergodicity for the observed behavioral measure and the cognitive model parameters persisted across different analytical choices.

In all, we examined 4 different strategies of performing between- and within-subjects analysis of how 1 observed behavioral measure and 3 latent cognitive parameters related to brain activity. In each of these 16 comparisons, within- and between-subjects inferences about brain-behavior associations diverged. This consistent finding across multiple analytical approaches and measures provides robust evidence that the neurocognitive dynamics of inhibitory control are fundamentally nonergodic.

Robustness of within-subjects associations to the structure of the computational model

Our primary analysis used the PRAD model of ref. [7] to learn 3 latent cognitive parameters (SSRT, probability of proactivity, and proactive delaying). We then used several control models to learn the same parameters, and for each of the 3 parameters, we compared the brain maps of within-subjects associations obtained using the PRAD model and using the control models (**SI Figure S6**). We found that each parameter's within-subjects associations were highly similar across PRAD and the control models, and thus that the within-subjects associations of these novel behavioral measures were robust to the structure of the computational model used to identify them.

Robustness of group-aggregated within-subjects associations to the method of aggregation

Within-subjects results showed considerable heterogeneity across subjects (**SI Figure S2**). To summarize these distributions, we have reported their simple Cohen's d's throughout this paper. However, this simple method of aggregating within-subjects results does not account for heterogeneity across subjects of the variance of within-subjects results. So, to test the robustness of our simple aggregated within-subjects results, we compared them to aggregated results from a 2 stage random effects meta-analysis that considers heterogeneity in the variance of subject-level estimates[8]. We found very high similarity between these 2 methods of aggregating within-subjects results (**SI Figure S8**). The Pearson correlation between simple averages or Cohen's d's versus the corresponding aggregated results using the 2 stage random effects meta-analysis ranged from 0.987 to 0.998.

Stability of within-subjects associations under resampling

To validate our approach, we investigated the stability and detectability of within-subjects brain-behavior associations in smaller samples, leveraging our large dataset to test the reliability of our findings (**Figure 8**).

To do this, first, we bootstrap resampled the results at varying sample sizes and computed the correlation of these results across brain areas with the results in the full sample using various collections of brain areas. We sampled subjects with replacement, with the number sampled varying between $N = 25$ and the full sample, and then correlated the effect sizes in the resamples with those in the full sample over the areas in each set of networks or regions, with strong correlations demonstrating stability of results across networks and regions. The within-subjects results in samples as small as 25 subjects correlated strongly with the results in the full sample.

Second, we directly examined the distributions of the effect sizes in the resampled data for regions of interest to gain another perspective on the reliability of the within-subjects findings. These finer-grained consistency checks showed that key results were observed frequently in small and moderate samples. For example, proactivity, as measured by either proactive parameter, was associated with right anterior insula suppression in greater than 95% of $N = 25$ samples, while proactivity's association with activation in the left retrosplenial cortex required $N \sim 100$ to emerge in greater than 95% of samples. However, some results showed considerable variability in the direction of effects for samples of $N > 100$, for example SSRT in the right anterior insula and proactive delaying in the right retrosplenial cortex.

We note that while our study's large number of subjects is important for assessing the stability and generalizability of our inferred within-subjects relationships, the power to detect a particular subject's relationship depends not on the number of subjects but on the number of temporal observations per subject (in our case, the number of trials)[9]. Thus, within-subjects research benefits from datasets that are large-scale in the number of subjects as well as large-scale in the number of time points collected.

Overall, these analyses demonstrate that within-subjects associations between brain activity and our model parameters (SSRT, probability of proactivity, and proactive delaying) were stable and detectable even with modest sample sizes. While some effects required larger samples to emerge consistently, many key findings were prevalent even in small samples. This stability supports the validity of our within-subjects approach and suggests that meaningful insights into neurocognitive mechanisms can be gained from studies with more modest sample sizes.

II. Supplementary Methods

Further details of PRAD model

The following priors were used for the PRAD model: $\delta_G \sim \text{Uniform}(0.0001{,}12)$. $\alpha_{G1} \sim \text{Uniform}(0.5{,}4)$. $\alpha_{G2} \sim \text{Uniform}(0.5{,}4)$. $\kappa_0 \sim \text{Normal}(0.1, \text{precision} = 0.3)$ truncated to (0,0.3). $\beta_G \sim \text{Beta}(1{,}1)$ truncated to (0.0001,0.9999). $\delta_S = p/\alpha_S$, where $p \sim \text{Uniform}(4{,}24)$ and $\alpha_S \sim \text{Uniform}(0.5{,}6)$. $\theta_0 \sim \text{Normal}(0, \text{precision} = 0.3)$. $\theta_1 \sim \text{Normal}(0, \text{precision} = 0.3)$. $\mu \sim \text{Beta}(0.5{,}0.5)$ truncated to (0.8,0.9999). $\gamma_1 \sim \text{Normal}(0, \text{precision} = 0.3)$. $\gamma_0 \sim \text{Uniform}(0{,}12)$. $\tau_G \sim \text{Uniform}\left(0.0001, \min(0.1, \text{RT}^*)\right)$, where RT^* is the reaction time after censoring for extremely small values. $\tau_S \sim \text{Uniform}(0.0001, \tau_G)$. Model inference was carried out using MCMC sampling, with the following hyperparameters: 4 chains with 30000 samples each. For each chain, we had a burn-in of 10000 samples, and a thinning factor of 10 was used to record 2000 of the remaining 20000 samples. Convergence was assessed and implemented using the methods outlined in ref. [7], checking for R-hat <= 1.1, utilizing convergent samples based on models with 2, 3, or 4 chains, and conducting robustness checks on sampling hyperparameters to ensure that this did not have an effect on the results. For model fitting, reaction-times less than 0.05s and greater than 1s (the experimental response window) were censored.

Measuring Simpson's paradox of brain networks

For each of SSRT, probability of proactivity, and proactive delaying, and for each of the Shirer networks, we computed bootstrap distributions of a measure of Simpson's paradox: we drew 10,000 samples of n subjects with replacement; for each resample, using the resample's between- and within-subjects associations, we computed the fraction of subjects whose (within-subjects) brain association with the parameter had the opposite sign of the between-subjects brain association with the parameter, in the network; then, we computed the mean and 95% confidence interval over the resamples of the fraction of opposite signs (SSRT and probability of proactivity $N = 4423$; proactive delaying $N = 4137$). The between-subjects association was the Pearson correlation between correct stop versus correct go activation and the subject-average parameter, which was recomputed for the subjects in each resample. The 95% confidence interval was calculated as the interval covering the 2.5th to 97.5th percentiles of the 10,000 fractions of opposite signs. The goal of using bootstrapping was to account for the strength of the between-subjects results. Next, the degree of Simpson's Paradox of each network was defined as the three-dimensional vector whose ith coordinate was the mean (over bootstrap resamples) fraction of subjects with opposite signs in the network for the ith cognitive model parameter. Then, the Euclidean distances were computed between the vectors and hierarchical clustering was performed on the distances using the linkage function in Scipy's cluster subpackage, hierarchy module with "method" set to "average".

PRAD-IR, PRAD-G, and RVM control models

PRAD-IR model. The PRAD-IR model is identical to the PRAD model, but was fit to the data from each of the two SST runs independently.

PRAD-G model. In the PRAD model, the dynamic drift rate on each trial depends on the duration of the go stimulus ($stim_t$). On go trials, since this is confounded with the go RT, it can be argued that this formulation is discriminative rather than a strictly generative model. To test the robustness of PRAD, we also test a fully generative model (PRAD-G) where the dynamic drift rate is only applied on stop trials (where the duration of the go stimulus is censored by the onset of the stop signal and $stim_t$ is not confounded with RT), while on go trials the drift rate is $\delta_0\ \mathbb{S}_{LR,t}$, similar to previous work[10]. All major behavioral results also replicate for this model.

RVM. The random variability model (RVM) is a simplification of the PRAD model without the dynamic hierarchical components and is equivalent to a full Bayesian implementation of the traditional horse-race model[11], but with the addition of allowing SSRT to vary randomly across trials, based on the parameters of a stopping drift diffusion process. It can also be considered a nested version of PRAD with the following constraints applied, plus a change in some priors: Constraints: $\alpha_{G1} = \alpha_{G2}$, $\delta_{G,t} = \delta_0\ \mathbb{S}_{LR,t}$, $\lambda_t = 0$, $\rho_t = 0$, $\beta_{S,t} = 0.5$.

Subject lists. For each of these 3 models, the subject list for brain-behavior association analyses was obtained by excluding from the primary subject list ($N = 4423$, described in the "inclusion criteria" subsection of the main methods section) the subjects who were not successfully fit by the model. Further, for analyses involving proactive delaying, subjects were additionally excluded if they had no trials with probability of proactivity greater than 0.5 during at least one run. This resulted in the following sample sizes: PRAD-IR SSRT and probability of proactivity $N = 3478$; PRAD-IR proactive delaying $N = 3160$; PRAD-G SSRT and probability of proactivity $N = 3913$; PRAD-G proactive delaying $N = 3619$; RVM SSRT $N = 3998$.

2 stage random effects meta-analysis

We fit the model

$$b_i = \gamma_{00} + u_i + r_i,$$

where b_i is subject i's observed result, γ_{00} is the unknown group-level result, u_i is a random effect modeling subject i's deviation from the group-level result ($\gamma_{00} + u_i$ is the true subject-level result), and r_i is the sampling error modeling the deviation of the observed b_i from the true subject-level result[8]. $u_i \sim N(0, \tau^2)$ and $r_i \sim N(0, V_i)$. In our case, the b_i are regression coefficients from fMRI general linear models, and the V_i are the standard errors squared of those coefficients. We fit the model with PyMARE's DerSimonianLaird.

We compared the similarity of the mean of the b_i with $\hat{\gamma}_{00}$, and the Cohen's d of the b_i with the 2 stage random effects meta-analysis Cohen's d, defined as $\hat{\gamma}_{00} / \left(\hat{\tau}^2 + \overline{V_i}\right)^{1/2}$, where $\overline{V_i}$ is the mean of the V_i and $\hat{\gamma}_{00}$ and $\hat{\tau}^2$ are our estimates of γ_{00} and τ^2. We examined the similarity for each brain voxel, except for voxels that had $V_i = 0$ for 1 or more subjects.

III. Supplementary Figures

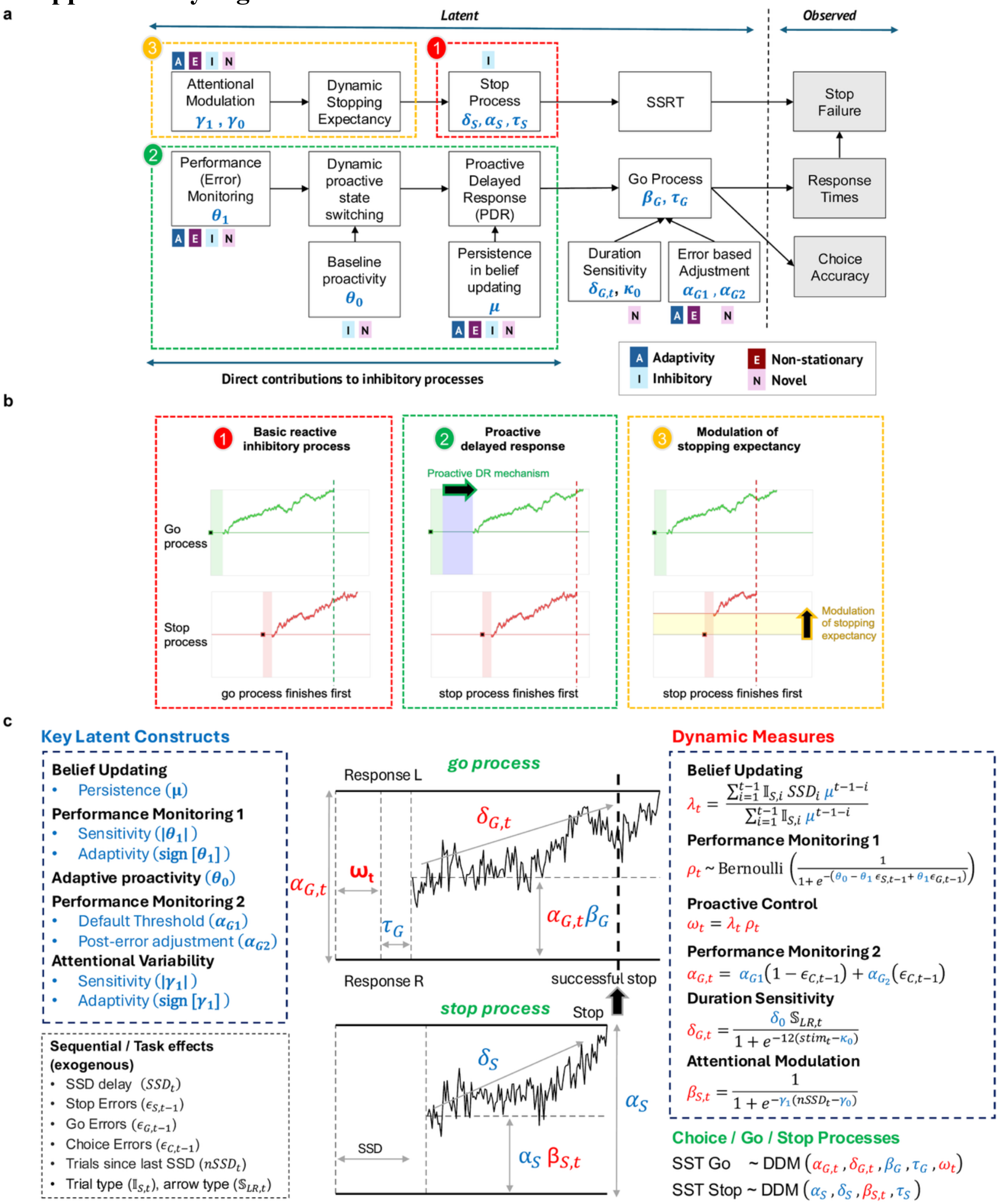

Supplementary Figure S1. The PRAD cognitive model. a, The PRAD model infers latent variables for each subject from their observed Go and Stop failure rates, response times, and choice accuracy. The latent variables relate to three mechanisms of dynamic inhibitory control: the basic reactive inhibitory process (red 1), proactive delaying of responses (green 2), and modulation of stopping expectancy (yellow 3). **b,** Visualization of the three mechanisms of dynamic inhibitory control. **c,** Mathematical details of how the model parameterizes the go and stop processes.

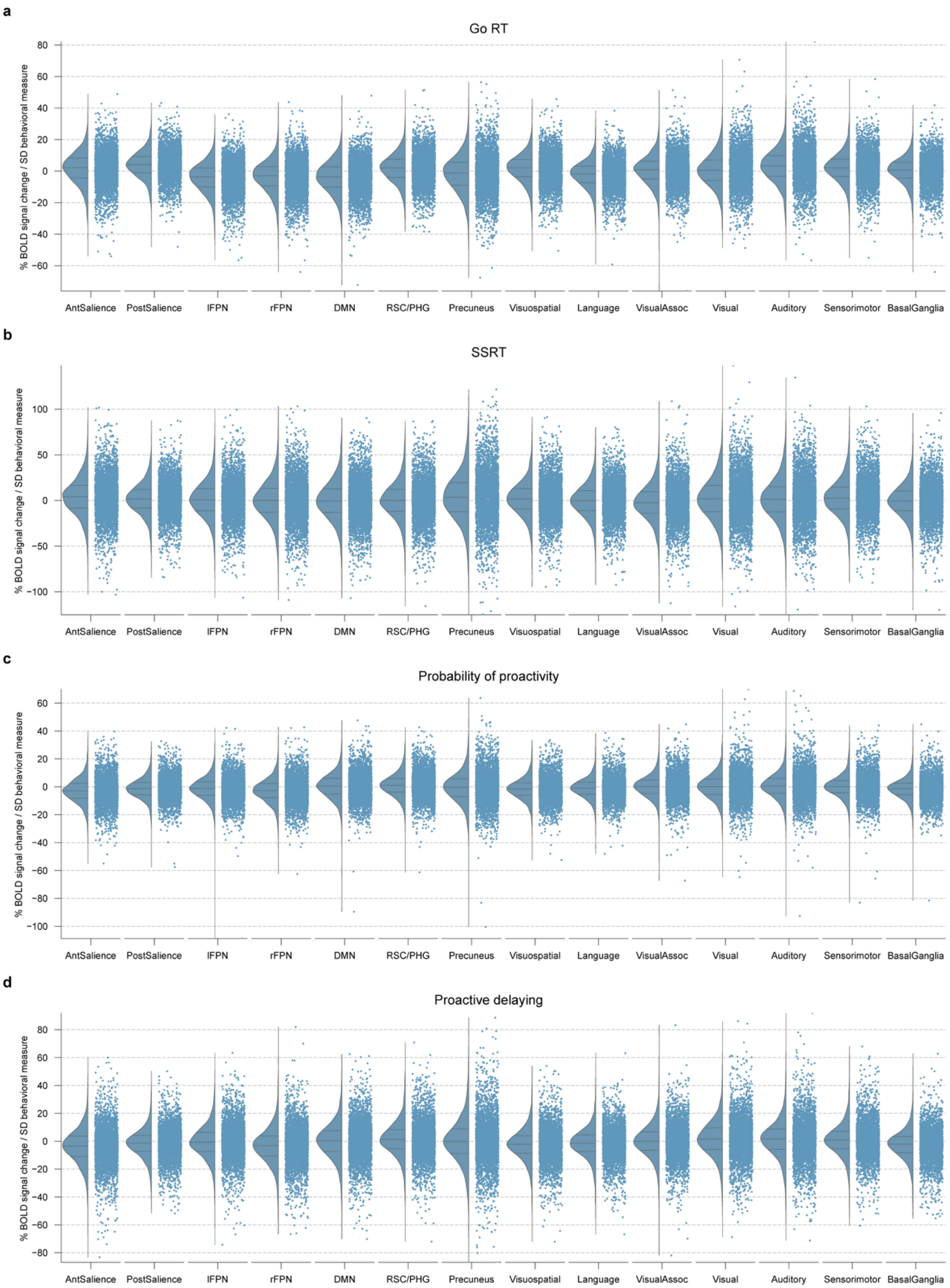

Supplementary Figure S2. Distributions over subjects of within-subjects associations. a-d, Distributions across subjects are shown for the within-subjects associations with brain activity of

behavioral measures: Go RT **(a)**, SSRT **(b)**, probability of proactivity **(c)**, and proactive delaying **(d)**. Dashed lines in the half-violin plots indicate the 25th, 50th, and 75th percentiles, and dots denote individual subjects.

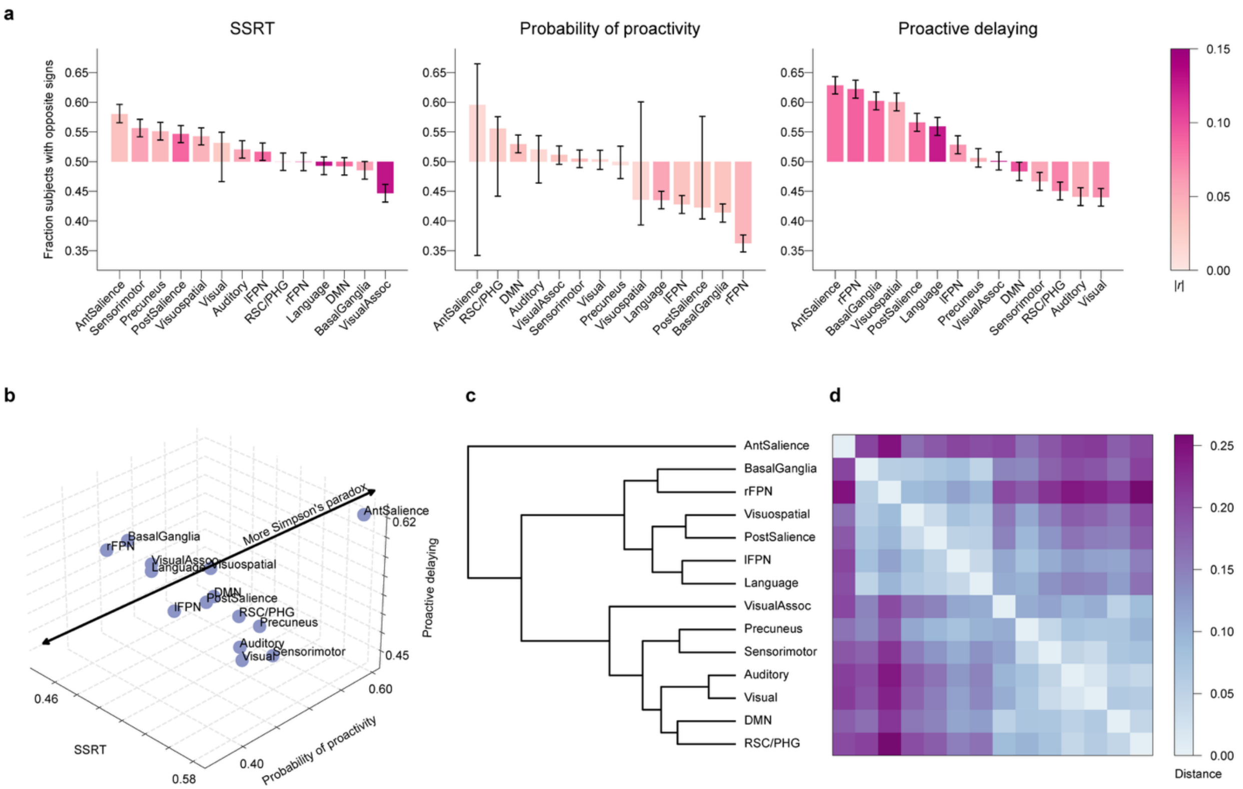

Supplementary Figure S3. Simpson's paradox across brain networks. a, Degree of Simpson's paradox exhibited in brain networks. Bar plots show the degree of Simpson's paradox for each brain network across three cognitive model parameters (SSRT, probability of proactivity, proactive delaying). The height of the bars is the mean fraction of subjects showing opposite-sign associations compared to between-subjects results. The bars are shaded by the magnitude of the between-subjects correlations. Error bars show 95% bootstrap confidence intervals. **b,** 3-dimensional embedding of each network. Networks are represented as points based on their degree of Simpson's paradox across the three parameters. **c-d,** Hierarchical clustering of networks based on their Simpson's paradox profiles. **c,** Dendrogram of clustering. **d,** Euclidean distances between network embeddings. Brain networks exhibited varying degrees of Simpson's paradox. The anterior salience network showed the highest degree of Simpson's paradox and a unique profile of this phenomenon distinct from that of all other networks.

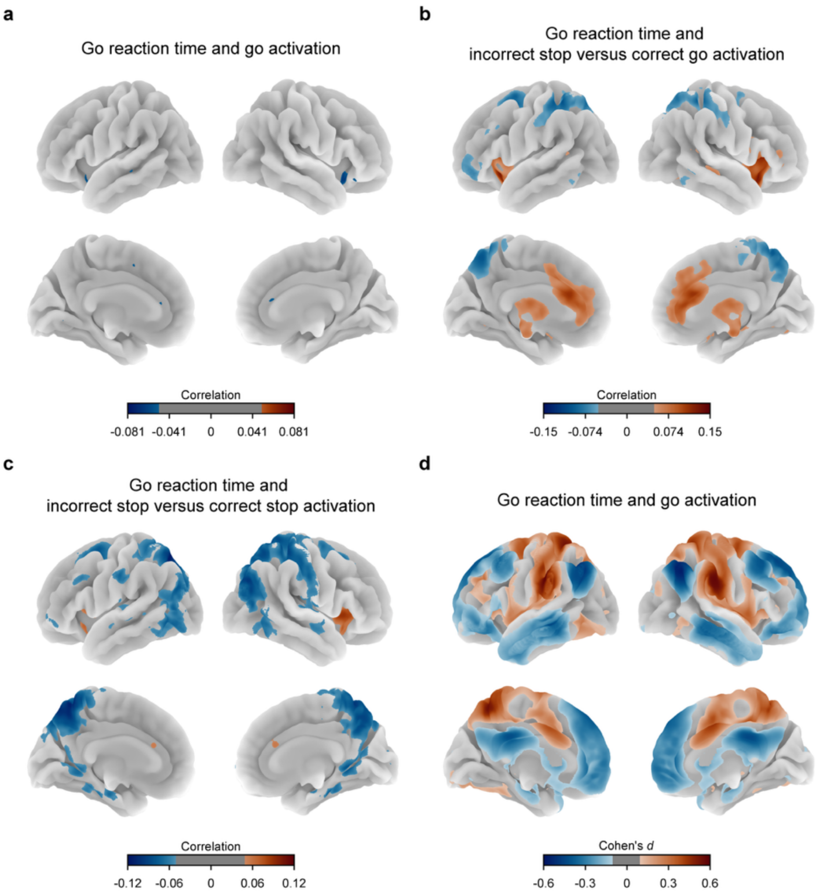

Supplementary Figure S4. Nonergodicity of observed RT was robust to type of between-subjects analysis. a-c, Between-subjects correlation maps of associations between go reaction time and go activation **(a)**, incorrect stop versus correct go activation **(b)**, and incorrect stop versus correct stop activation **(c)**. In each voxel, subject-average go reaction time was correlated with subject-average brain activation. The correlation maps were thresholded at Pearson $r \geq 0.05$. **d,** Within-subjects Cohen's d map of associations between go reaction time and brain activity. For each subject and in each voxel, brain activity was regressed on reaction time on go trials; the Cohen's d's of the regression coefficients were then calculated. The resulting Cohen's d map was thresholded at Cohen's $d \geq 0.1$.

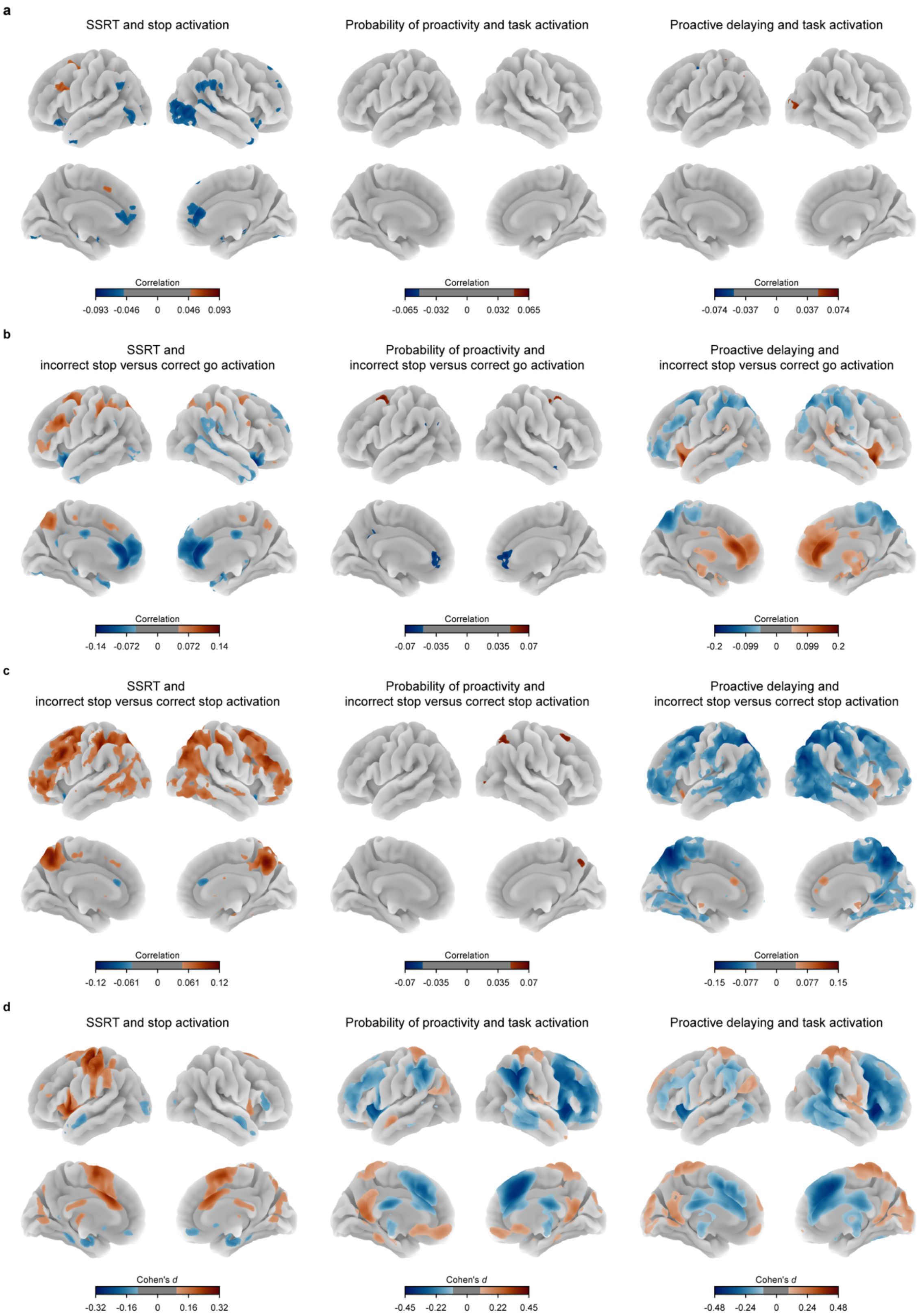

Supplementary Figure S5. Nonergodicity of computational model parameters was robust to type of between-subjects analysis. a-c, Between-subjects correlation maps of associations

between SSRT and stop activation, probability of proactivity and task activation, and proactive delaying and task activation **(a)**; and between-subjects correlation maps of associations between the cognitive model parameters and incorrect stop versus correct go activation **(b)**, and incorrect stop versus correct stop activation **(c)**. In each voxel, subject-average brain activation was correlated with subject-average SSRT, probability of proactivity, and proactive delaying. The correlation maps were thresholded at ≥ 0.05. **d,** Within-subjects Cohen's d maps of associations between cognitive model parameters and brain activity. For each subject and in each voxel, brain activity was regressed on: SSRT on stop trials, probability of proactivity on all trials, and proactive delaying on all trials. The resulting Cohen's d maps were thresholded at ≥ 0.1.

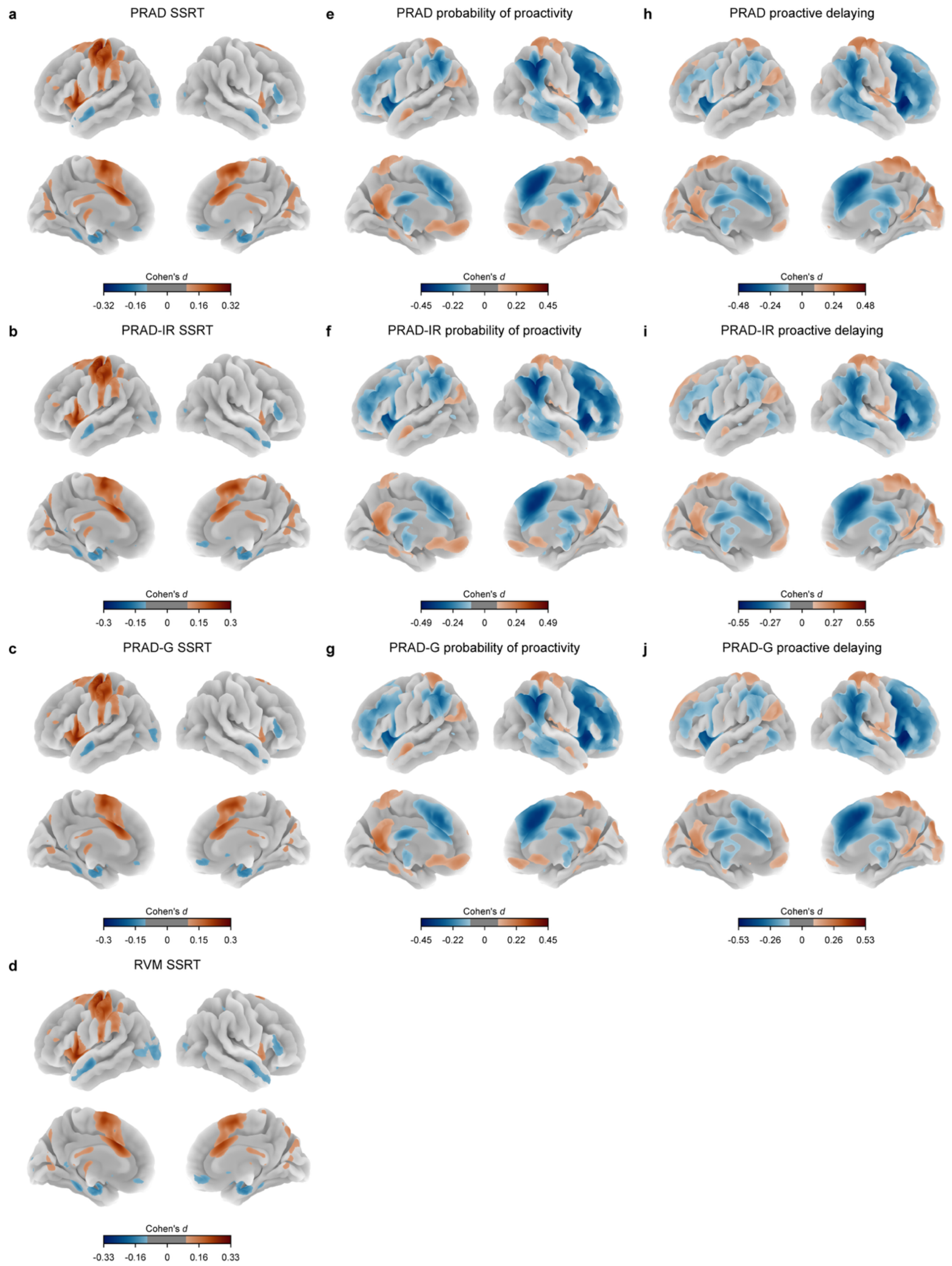

Supplementary Figure S6. Within-subjects associations were robust to PRAD modeling assumptions. a-d, Within-subjects Cohen's d maps for SSRT inferred by PRAD and 3 control

models: the PRAD model **(a)**, PRAD fit independently to each SST run (PRAD-IR) **(b)**, a version of PRAD that does not account for drift rate variability on go trials (PRAD-G) **(c)**, and a model that assumes variability in trial-level SSRT but without the attentional and proactive mechanisms (RVM) **(d)**. **e-g,** Within-subjects Cohen's d maps for probability of proactivity inferred by PRAD and 2 control models: PRAD **(e)**, PRAD-IR **(f)**, and PRAD-G **(g)**. **h-j,** Within-subjects Cohen's d maps for proactive delaying inferred by PRAD and 2 control models: PRAD **(h)**, PRAD-IR **(i)**, and PRAD-G **(j)**. The Cohen's d maps were thresholded at Cohen's $d \geq 0.1$. PRAD SSRT and probability of proactivity $N = 4423$; PRAD proactive delaying $N = 4137$; PRAD-IR SSRT and probability of proactivity $N = 3478$; PRAD-IR proactive delaying $N = 3160$; PRAD-G SSRT and probability of proactivity $N = 3913$; PRAD-G proactive delaying $N = 3619$; RVM SSRT $N = 3998$.

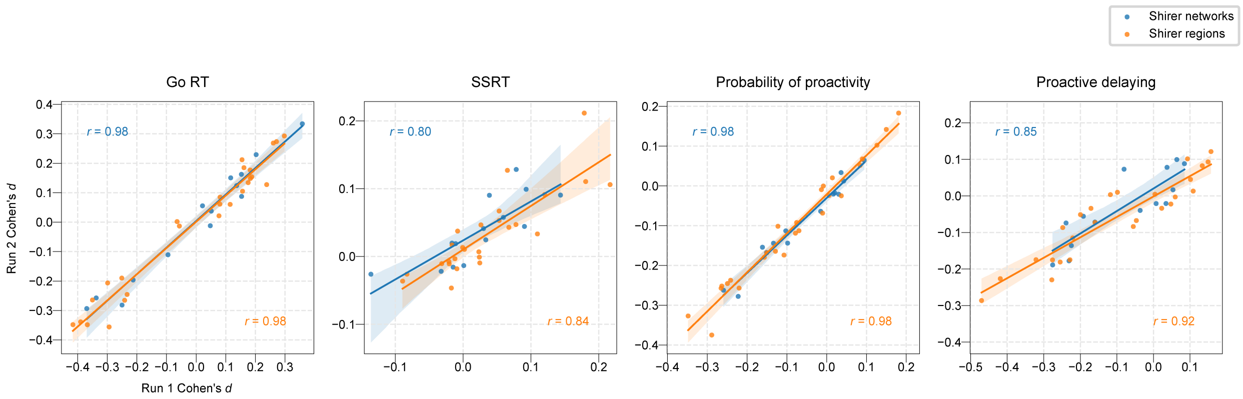

Supplementary Figure S7. Within-subjects associations were robustly observed between task runs. Group-average within-subjects brain-behavior associations were reliably observed across the 2 SST runs. SSRT, probability of proactivity, and proactive delaying were obtained from the PRAD model fit to each of the SST runs independently. Brain activity was regressed on each behavioral measure, and associations were extracted in 2 brain parcellations (the Shirer networks and regions). Dots denote brain areas and the shaded areas show 95% bootstrap confidence intervals for the regression lines. Go RT $N = 4423$; SSRT and probability of proactivity $N = 3478$, proactive delaying $N = 3160$.

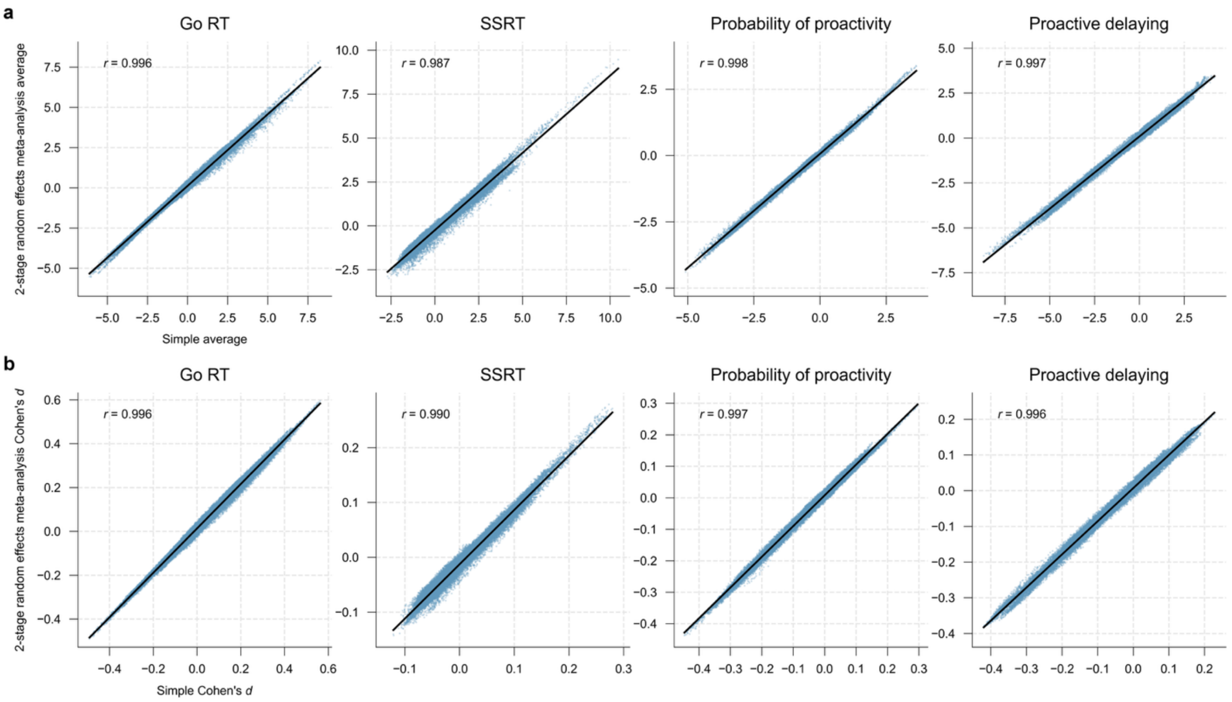

Supplementary Figure S8. Aggregation of within-subjects associations was robust to use of 2-stage random effects meta-analysis. a-b, Aggregated results from 2-stage random effects meta-analysis were compared to simple aggregated results: average **(a)** and Cohen's d **(b)**. The results that were aggregated were the distributions across subjects of the within-subjects brain-behavior associations for Go RT, SSRT, probability of proactivity, and proactive delaying. The 2-stage random effects meta-analysis accounted for between-subjects heterogeneity in the precision of within-subjects estimation. The simple average was the mean and the simple Cohen's d was the mean divided by the standard deviation. Dots denote voxels. Go RT, SSRT, and probability of proactivity $N = 4423$; proactive delaying $N = 4137$.

Supplementary References

1. Li, C. S. *et al.* Neural correlates of impulse control during stop signal inhibition in cocaine-dependent men. *Neuropsychopharmacology* **33**, 1798-1806, doi:10.1038/sj.npp.1301568 (2008).
2. Li, C. S., Huang, C., Constable, R. T. & Sinha, R. Imaging response inhibition in a stop-signal task: neural correlates independent of signal monitoring and post-response processing. *J Neurosci* **26**, 186-192, doi:10.1523/JNEUROSCI.3741-05.2006 (2006).
3. Chevrier, A. & Schachar, R. J. BOLD differences normally attributed to inhibitory control predict symptoms, not task-directed inhibitory control in ADHD. *J Neurodev Disord* **12**, 8, doi:10.1186/s11689-020-09311-8 (2020).
4. Chaarani, B. *et al.* Baseline brain function in the preadolescents of the ABCD Study. *Nat Neurosci* **24**, 1176-1186, doi:10.1038/s41593-021-00867-9 (2021).
5. Cai, W. *et al.* Hyperdirect insula-basal-ganglia pathway and adult-like maturity of global brain responses predict inhibitory control in children. *Nature communications* **10**, 4798 (2019).
6. Aron, A. R. & Poldrack, R. A. Cortical and subcortical contributions to stop signal response inhibition: role of the subthalamic nucleus. *Journal of Neuroscience* **26**, 2424-2433 (2006).
7. Mistry, P. K., Warren, S. L., Branigan, N. K., Cai, W. & Menon, V. Computational Modeling of Proactive, Reactive, and Attentional Dynamics in Cognitive Control. *bioRxiv*, 2024.2010. 2001.615613 (2024).
8. Lee, S. A. & Gates, K. M. From the individual to the group: Using idiographic analyses and two-stage random effects meta-analysis to obtain population level inferences for within-person processes. *Multivariate Behavioral Research* **59**, 1220-1239 (2024).
9. Fisher, A. J., Medaglia, J. D. & Jeronimus, B. F. Lack of group-to-individual generalizability is a threat to human subjects research. *Proc Natl Acad Sci U S A* **115**, E6106-E6115, doi:10.1073/pnas.1711978115 (2018).
10. Weigard, A., Matzke, D., Tanis, C. & Heathcote, A. A cognitive process modeling framework for the ABCD study stop-signal task. *Developmental Cognitive Neuroscience* **59**, 101191 (2023).
11. Band, G. P., Van Der Molen, M. W. & Logan, G. D. Horse-race model simulations of the stop-signal procedure. *Acta psychologica* **112**, 105-142 (2003).
