# Supplementary material for: Nonergodicity and Simpson’s paradox in neurocognitive dynamics of cognitive control
Source: Nat Commun. 2026 Apr 27;17:3494. doi: 10.1038/s41467-026-71404-0 (PMC13121725; doi:10.1038/s41467-026-71404-0)
Supplement: Supplementary file 2 — Reporting Summary [file 41467_2026_71404_MOESM2_ESM.pdf]

nature portfolio

Corresponding author(s): Percy K. Mistry, Nicholas K. Branigan, Vinod Menon

Last updated by author(s): Feb. 3, 2026

Reporting Summary

Nature Portfolio wishes to improve the reproducibility of the work that we publish. This form provides structure for consistency and transparency in reporting. For further information on Nature Portfolio policies, see our Editorial Policies and the Editorial Policy Checklist.

Statistics

For all statistical analyses, confirm that the following items are present in the figure legend, table legend, main text, or Methods section.

n/a	Confirmed	
☐	☒	The exact sample size (n) for each experimental group/condition, given as a discrete number and unit of measurement
☐	☒	A statement on whether measurements were taken from distinct samples or whether the same sample was measured repeatedly
☐	☒	The statistical test(s) used AND whether they are one- or two-sided<br>*Only common tests should be described solely by name; describe more complex techniques in the Methods section.*
☐	☒	A description of all covariates tested
☐	☒	A description of any assumptions or corrections, such as tests of normality and adjustment for multiple comparisons
☐	☒	A full description of the statistical parameters including central tendency (e.g. means) or other basic estimates (e.g. regression coefficient) AND variation (e.g. standard deviation) or associated estimates of uncertainty (e.g. confidence intervals)
☐	☒	For null hypothesis testing, the test statistic (e.g. F, t, r) with confidence intervals, effect sizes, degrees of freedom and P value noted<br>*Give P values as exact values whenever suitable.*
☐	☒	For Bayesian analysis, information on the choice of priors and Markov chain Monte Carlo settings
☒	☐	For hierarchical and complex designs, identification of the appropriate level for tests and full reporting of outcomes
☐	☒	Estimates of effect sizes (e.g. Cohen's d, Pearson's r), indicating how they were calculated

Our web collection on statistics for biologists contains articles on many of the points above.

Software and code

Policy information about availability of computer code

Data collection	This is described in other work (Casey et al., 2018).
Data analysis	All code used in this study has been archived at https://doi.org/10.5281/zenodo.18626601. For details on the software used, see the "Software" subsection of the "Methods" section.<br><br>Data were processed and analyzed using Python (version 3.9.16), Scipy (version 1.11.4), Seaborn (version 0.13.2), Nilearn (version 0.10.1), FSL FLIRT (version 6.0), MATLAB (version R2020b), PyMARE (version 0.0.10), and JAGS (version 4.3.0). Brain maps used the vik colormap.

For manuscripts utilizing custom algorithms or software that are central to the research but not yet described in published literature, software must be made available to editors and reviewers. We strongly encourage code deposition in a community repository (e.g. GitHub). See the Nature Portfolio guidelines for submitting code & software for further information.

Data

Policy information about availability of data

All manuscripts must include a data availability statement. This statement should provide the following information, where applicable:

- Accession codes, unique identifiers, or web links for publicly available datasets
- A description of any restrictions on data availability
- For clinical datasets or third party data, please ensure that the statement adheres to our policy

Data used in this study were from the ABCD study (https://abcdstudy.org/), held in the National Institute of Mental Health Data Archive. These data are available to

eligible researchers.

Research involving human participants, their data, or biological material

Policy information about studies with human participants or human data. See also policy information about sex, gender (identity/presentation), and sexual orientation and race, ethnicity and racism.

Reporting on sex and gender	We did not investigate sex differences.
Reporting on race, ethnicity, or other socially relevant groupings	We did not investigate race, ethnicity, or other socially relevant groupings.
Population characteristics	The participants were 9-10 year-old children.
Recruitment	We only performed secondary data analysis.
Ethics oversight	We only performed secondary data analysis.

Note that full information on the approval of the study protocol must also be provided in the manuscript.

Field-specific reporting

Please select the one below that is the best fit for your research. If you are not sure, read the appropriate sections before making your selection.

☒ Life sciences ☐ Behavioural & social sciences ☐ Ecological, evolutionary & environmental sciences

For a reference copy of the document with all sections, see nature.com/documents/nr-reporting-summary-flat.pdf

Life sciences study design

All studies must disclose on these points even when the disclosure is negative.

Sample size	The ABCD study recruited 11,880 children. The analyses in this paper used data from all subjects for whom data was available and passed inclusion criteria (N=4,423 or N=4,137 for analyses involving the proactive delaying measure). For the 4,423 subjects, sex assigned at birth was male for 2,111 and female for 2,312. In this paper, the participants were 9-10 years old. Both the children and their guardians were compensated. Informed consent was obtained from all participants.
Data exclusions	Data were from the baseline visit of the ABCD study (Collection #2573), N=11817. Approval was received from institutional review boards at the University of California San Diego and the study sites. Subjects were excluded if they did not meet each of the following criteria: meet the ABCD study's SST task-fMRI inclusion recommendations (in abcd_imgincl01.txt, imgincl_sst_include==1; N=3546 excluded); have 2 SST fMRI runs of good quality (in mriqcrp20301.txt, iqc_sst_total_ser==iqc_sst_good_ser==2; N=677 excluded); are successfully fit with the cognitive model of the SST (N=562 excluded); have enough volumes acquired to cover the SST experiment (the last SST trial must have happened no more than 2 seconds after the final volume was acquired; N=5 excluded); have mean framewise displacement of less than 0.5 mm for both runs (calculated using the method of ref. 67; N=2058 excluded); have release 4.0 minimally processed events.tsv files of shape (181,3) for both runs (N=7 excluded); and have consistent release 4.0 behavioral data (in release 4.0, for some subjects, the "sst.csv" files from ABCD Task fMRI SST Trial Level Behavior, abcd_sst_tlb01, disagreed with the minimally processed "events.tsv" files; for example, one trial might be labeled a go trial by one file and a stop trial by the other; N=102 excluded). Then, we excluded siblings by randomly keeping one member from each family (using the genetic_paired_subjectid variables from gen_y_pihat; N=426 excluded) and excluded subjects without scanner serial number recorded (in mri_y_adm_info, missing mri_info_deviceserialnumber; N=11 excluded). Applying these inclusion criteria left us with a sample of N=4423. For analyses involving the proactive delaying, a further 286 subjects were excluded who had no trials with probability of proactivity greater than 0.5 during at least one run, and therefore, by definition, a proactive delaying of 0 for all trials of at least one run. For these subjects, we were unable to examine within-subjects relationships between proactive delaying and brain activity. To maintain comparability of the between- and within-subjects analyses, we also excluded these subjects from the between-subjects analyses involving proactive delaying. Thus, analyses involving the proactive delaying used a sample of N=4137.
Replication	Results were resampled at sample sizes between N=25 and N=4,423 / N=4,137. The results of this analysis suggest the replicability of the overall findings.
Randomization	Randomization was not used. The MRI scanner used for data acquisition was regressed out of the results. Other covariates were not hypothesized as confounding the results.
Blinding	There were no experimental groups, so blinding is not applicable.

Reporting for specific materials, systems and methods

We require information from authors about some types of materials, experimental systems and methods used in many studies. Here, indicate whether each material, system or method listed is relevant to your study. If you are not sure if a list item applies to your research, read the appropriate section before selecting a response.

Materials & experimental systems

n/a	Involved in the study
☒	☐ Antibodies
☒	☐ Eukaryotic cell lines
☒	☐ Palaeontology and archaeology
☒	☐ Animals and other organisms
☒	☐ Clinical data
☒	☐ Dual use research of concern
☒	☐ Plants

Methods

n/a	Involved in the study
☒	☐ ChIP-seq
☒	☐ Flow cytometry
☐	☒ MRI-based neuroimaging

Plants

Seed stocks

Report on the source of all seed stocks or other plant material used. If applicable, state the seed stock centre and catalogue number. If plant specimens were collected from the field, describe the collection location, date and sampling procedures.

Novel plant genotypes

Describe the methods by which all novel plant genotypes were produced. This includes those generated by transgenic approaches, gene editing, chemical/radiation-based mutagenesis and hybridization. For transgenic lines, describe the transformation method, the number of independent lines analyzed and the generation upon which experiments were performed. For gene-edited lines, describe the editor used, the endogenous sequence targeted for editing, the targeting guide RNA sequence (if applicable) and how the editor was applied.

Authentication

Describe any authentication procedures for each seed stock used or novel genotype generated. Describe any experiments used to assess the effect of a mutation and, where applicable, how potential secondary effects (e.g. second site T-DNA insertions, mosiacism, off-target gene editing) were examined.

Magnetic resonance imaging

Experimental design

Design type

Event-related task design.

Design specifications

Each subject performed 2 runs of the stop signal task. Each run consisted of 180 trials, of which 30 were stop trials and the remainder were go trials. Each trial lasted 1 second. Inter-trial intervals were 1-2 seconds.

Behavioral performance measures

The trial types used were correct go, incorrect go, correct late go, incorrect late go, no response go, correct stop, incorrect stop, and SSD stop. Subjects were excluded from analysis if they had: <60% correct go trials, >30% incorrect go trials, >30% late go trials, >30% no response go trials, correct go response time < incorrect stop response time, stop trial accuracy <20%, or stop trial accuracy >80%.

Acquisition

Imaging type(s)

Functional

Field strength

3T

Sequence & imaging parameters

These are described in other work (Casey et al., 2018).

Area of acquisition

Whole brain

Diffusion MRI ☐ Used ☒ Not used

Preprocessing

Preprocessing software

Minimal processing of the imaging data, performed by ABCD, included distortion correction and motion correction, and is described in other work (Hagler et al., 2019). We then further preprocessed that data using Nilearn (version 0.10.1) and FSL FLIRT (version 6.0).

Normalization

Linear spatial normalization to an echo-planar imaging template was performed using FSL FLIRT.

Normalization template

SPM12's toolbox/OldNorm/EPI.nii

Noise and artifact removal

6 motion parameters (translational and rotational displacement along each of three axes) and 6 cosine basis functions (corresponding to high-pass filtering at 0.01 Hz) were included as covariates in each first-level fMRI general linear model.

Volume censoring

Not performed

Statistical modeling & inference

Model type and settings

We combined the regression results from the 2 runs with a fixed effects model. Whole-brain results were thresholded based on effect sizes (Pearson r>0.05 and Cohen's d>0.1). Results in regions of interest used FDR correction over the regions of interest. For more details, see subsection "Between- and within-subjects, general linear model analysis of fMRI" in the "Methods" section.

Effect(s) tested

We tested the correlation between fMRI general linear model regression coefficients and behavioral measures, the mean of fMRI general linear model regression coefficients, and the correlations between within-subjects brain maps. See subsection "Statistical testing" in the "Methods" section.

Specify type of analysis: ☐ Whole brain ☐ ROI-based ☒ Both

Anatomical location(s): See subsection "Networks and regions of interest" in the "Methods" section.

Statistic type for inference (See Eklund et al. 2016)

Voxel-wise effect sizes are reported. In regions of interest, statistical testing uses permutation inference and FDR correction.

Correction

For region of interest results, FDR correction is performed.

Models & analysis

n/a	Involved in the study
☒	☐ Functional and/or effective connectivity
☒	☐ Graph analysis
☒	☐ Multivariate modeling or predictive analysis
